# dUTPase is essential in zebrafish development and possesses several single‐nucleotide variants with pronounced structural and functional consequences

**DOI:** 10.1002/2211-5463.70176

**Published:** 2025-12-16

**Authors:** Viktória Perey‐Simon, Angéla Békesi, Latifa Kazzazy, Jázmin Mihály, Máté Varga, Beáta G. Vértessy, Kinga Nyíri

**Affiliations:** ^1^ Department of Applied Biotechnology and Food Science, Faculty of Chemical Technology and Biotechnology Budapest University of Technology and Economics Hungary; ^2^ Institute of Molecular Life Sciences HUN‐REN Research Centre for Natural Sciences Budapest Hungary; ^3^ Department of Genetics ELTE Eötvös Loránd University Budapest Hungary

**Keywords:** development, dUTPase, natural mutation, protein stability, single‐nucleotide polymorphism, zebrafish

## Abstract

Genome stability and faithful DNA replication are essential for cell viability. Numerous interlinked pathways in DNA damage recognition, repair and maintenance of physiologically competent nucleotide pools contribute to providing a solid framework to uphold DNA integrity. The enzyme family of dUTPases is involved in balancing the appropriate nucleotide pools by removing dUTP from the cellular milieu and providing dUMP for thymidylate *de novo* biosynthesis. In the present study, we show that dUTPase is essential for normal development in zebrafish. We also found that the fish *dut* gene from different genomes contains several single‐nucleotide variations (SNPs). This observation prompted structural and functional investigations of the SNP variants at the protein level. Results indicated that none of the mutation sites of the variants are within the active site. Still, one of the variants showed drastically lower protein stability and catalytic efficiency as compared to the other two dUTPase variants, underlining the importance of detailed characterization of SNPs even at sites distant from the active site. In conclusion, we demonstrate the importance of dUTPase function in zebrafish development and unveil the role of several point mutations on protein structure and function.

## Graphical abstract

dUTPases are involved in balancing the appropriate nucleotide pools. We showed that dUTPase is essential for normal development in zebrafish. The different zebrafish genomes contain several single‐nucleotide variations (SNPs) of the *dut* gene. One of the dUTPase variants displayed drastically lower protein stability and catalytic efficiency as compared to the other two, underscoring the functional consequences of naturally occurring mutations.

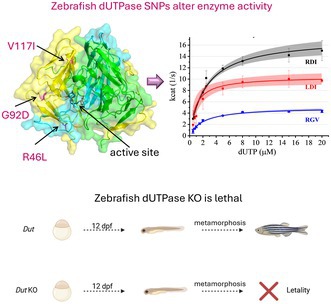

**Abbreviations**
CAGE: cap analysis of gene expression
dpf: days post fertilization
dUMP: deoxyuridine monophosphate
dUTPase: deoxyuridine‐5′‐triphosphate nucleotide hydrolase
GEO: gene expression omnibus
GO: genome ontology
IGV: integrated genome viewer
KO: knockout
SNPs: single‐nucleotide variations
TSSs: transcriptional start sites

Deoxyuridine‐5′‐triphosphate nucleotide hydrolase (dUTPase) catalyzes the hydrolysis of dUTP into deoxyuridine monophosphate (dUMP) and pyrophosphate. This enzymatic activity is critical for cellular viability, as it ensures a low intracellular dUTP/dTTP ratio, thereby preventing the misincorporation of uracil into DNA [1]. Since DNA polymerases do not discriminate between uracil and thymine during DNA synthesis, regulating this nucleotide pool by dUTPase plays a fundamental role in maintaining genomic integrity [2].

From the protein structural point of view, dUTPases—apart from a few viral and protozoan enzymes—are homotrimers [3, 4, 5, 6, 7, 8]. The substrate binding pocket of the homotrimeric dUTPases is formed on the interaction surface of two subunits, from which one subunit contributes with conserved motifs 1, 2, and 4, while the other subunit contributes with motif 3 [9, 10, 11]. The C‐terminal segment of the third subunit (motif 5) closes the active site to result in a catalytically competent conformation (Fig. S1) [12, 13].

Bacteria and archaea completely lacking dUTPase function have been found in nature [14]. The viability of these organisms may result from (i) reduced activity, absence, or inhibition of UNG; (ii) compensation by a promiscuous nucleotide pyrophosphatase exhibiting dUTPase activity; or (iii) the acquisition of dUTPase from bacteriophages. Despite these exceptions, in general, dUTPase activity is essential in prokaryotes. Mutations that reduce the activity of the *Escherichia coli* dUTPase enzyme and impairment of the *DUT* gene in *Mycobacterium smegmatis* were found to be lethal [15, 16]. Of note, *M. smegmatis* was found to be viable with an inactive dUTPase, but not with a fully active dUTPase lacking a species‐specific 5‐residue long loop, suggesting that in *Mycobacterium smegmatis* the dUTPase has additional essential functions [11, 16].

Similarly, in eukaryotes, silencing of the gene encoding dUTPase resulted in lethality in all tested species (*Caenorhabditis elegans*, *Dugesia ryukyuensis*, *Drosophila melanogaster* and *Arabidopsis thaliana*) [17, 18, 19, 20]. Furthermore, CRISPR/Cas9‐mediated knockout of dUTPase in mice leads to early embryonic lethality [21].

Notably, there is a known disease‐associated exon SNP in human dUTPase (Y54C), which is linked to a novel monogenic syndrome associated with early‐onset diabetes and bone marrow failure [22]. While this mutation does not significantly affect substrate binding, it has been shown to significantly decrease the thermal stability of the enzyme as well as its activity at body temperature [23]. The same mutation leads to thrombocytopenia (i.e., abnormally low platelet count in peripheral blood) in rabbits [24].

It has been reported that effective dUTPase silencing does not result in a phenotypic change in human cell lines [25, 26, 27], but still sensitizes these cells to cancer therapy. Nevertheless, the absence of any reported dUTPase knockout (KO) human cell lines to date suggests that the protein may also be essential in these models.

Vertebrate animal models have demonstrated their usefulness in various physiological and pathophysiological studies. In particular, the zebrafish (*Danio rerio*) represents a highly effective model for investigating both genetic and acquired human diseases, and can also help in drug discovery and development, as shown in numerous earlier studies [28, 29, 30, 31, 32, 33, 34, 35, 36]. Moreover, the zebrafish model has also gained prominence in cancer research, providing a versatile platform for studying tumor biology and evaluating therapeutic interventions [28, 37, 38, 39, 40].

A key advantage of this vertebrate model lies in the substantial conservation of its molecular and cellular mechanisms with those present in mammals, particularly those involved in development. The zebrafish genome shares approximately 70% of its genes with humans, enabling functional genomics studies in a vertebrate context [29]. Moreover, zebrafish are easy to maintain, require limited space, reproduce efficiently, and do not require complex breeding infrastructure, making them accessible for a wide range of laboratories.

One notable feature is the external fertilization and embryonic development of zebrafish, which permit direct observation and experimental manipulation of early developmental stages.

A single zebrafish can produce between 100 and 300 embryos every few weeks, facilitating high‐throughput studies in relatively small laboratory environments. They undergo rapid development, with embryogenesis typically completed within 5 days. Furthermore, both embryos and larvae are transparent, allowing detailed visualization of internal organogenesis and physiological processes [28].

Intriguingly, a detailed analysis of zebrafish Dut function has been hitherto missing. While some previous research has linked dUTPase expression to egg quality [41] and to the regeneration of various zebrafish tissues [42, 43], no detailed analysis of the gene exists. Furthermore, given the importance of the well‐characterized maternal‐zygotic transition in the development of this species [44, 45] and the possible involvement of noncanonical DNA modification during the activation of the genome [46], zebrafish embryogenesis could provide an important model to understand and characterize dUTPase function. Genome databases show several single‐point variations (SNPs) in the zebrafish *dut* gene, also necessitating investigations into whether these mutations affect protein structure and function.

To expand our understanding of this well‐established model organism in developmental genetics, we aimed to characterize the zebrafish dUTPase protein variants and the effect of *dut* loss of function on the viability of this animal.

## Methods

### *In silico* analysis of the *Danio rerio* dUTPase and its mutants

Structural models were generated by AlphaFold3 [47]. Various prediction servers were used to assess the effect of the mutations [48, 49, 50, 51, 52, 53, 54].

### Plasmid cloning

The gene encoding the mitochondrial dUTPase of *Danio rerio* (UniProt ID: Q5XJ23) was ordered from GeneUniversal, cloned in the pET‐15b vector in frame with the N‐terminal His6‐tag and thrombin recognition and cleavage site, The protein produced from this vector is denoted as *Dr*DUT‐mito.

Deletion of the mitochondrial leader sequence (residues 1–26) at the N terminus of the mitochondrial dUTPase of *Danio rerio* (UniProt ID: Q5XJ23) was performed by mutagenesis (Primer 1 and 2 in Table S1). The protein expressed from this vector is denoted as *Dr*DUT‐RGV.

Double mutation was made in the plasmid encoding *Dr*DUT‐RGV to obtain the expression vector of a *Dr*DUT‐RDI, that is, a protein having the same variations as the isoform with UniProt ID: A0A8M9PI68 (G92D and V117I in Q5XJ23). A Megaprimer coding the G92D and V117I mutations was first made by PCR (Primer 3 and 4 in Table S1). The resulting PCR product (i.e., the megaprimer) was purified from a 1% agarose gel with the NucleoSpin Gel and PCR CleanUp Kit. For the second PCR, we used the purified megaprimer and Primer 5 (cf. Table S1).

To create *Dr*DUT‐LDI, the variant corresponding to UniProt entries A0A8M9P120 and A0A8M9PA15 the plasmid encoding *Dr*DUT‐RDI was subjected to mutagenesis (Primer 6 and 7 Table S1) resulting in a change of arginine 46 to leucine.

The resulting constructs were subjected to KLD treatment and then were transformed into *E. coli* XL1Blue strain, which were first grown on solid TC, CAR LB agar plates then single clones were grown in liquid TC, CAR LB. The plasmids were purified with Macherey‐Nagel NucleoSpin Plasmid CleanUp Kit; the concentration was measured by Nanodrop at 260 nm. All constructs were checked by Sanger sequencing.

### Protein expression

The proteins were expressed in the *Escherichia coli* (*E. coli*) Rosetta strain in LB media. The proteins were overexpressed upon induction with 0.5 mm isopropyl‐β‐*d*‐thiogalactopyranoside (IPTG) at OD_600_ = 0.6 for 4 h at 37 °C. The pellets were lysed in lysis buffer (50 mm Tris pH 7.5, 300 mm NaCl, 0.5 mm EDTA, 5 mm benzamidine, 1 mm PMSF, 0.1 V/V% TritonX100, 10 mm 2‐mercaptoethanol, 10 mg·L^−1^ DNase and a half tablet of EDTA‐free protease inhibitor). The resulting suspensions were sonicated 3 × 1 min on ice with 1 min pause and then centrifuged at 15 000 ***g*** for 30 min at 4 °C. The supernatants were purified on a Ni‐NTA‐column. Briefly, the column was equilibrated with the lysis buffer, and then, the supernatant was loaded to the column. The column was washed with the LS (low salt buffer: 20 mm HEPES, pH 7.5, 30 mm KCl), HS (high salt buffer: 20 mm HEPES, pH 7.5, 300 mm KCl), and 50 mm imidazole LS buffer. The samples were eluted with 500 mm imidazole in LS buffer. The protein content of the fractions was followed with Bradford. We analyzed the protein fractions by SDS/PAGE and the most concentrated dUTPase fraction was dialyzed to 20 mm HEPES, pH 7.5, 100 mm NaCl, 5 mm MgCl_2_, 10 mm 2‐mercaptoethanol. The concentration of the dialyzed samples was measured by using UV absorbance on Nanodrop. Extinction coefficients were estimated based on sequence by Expasy (cf Table S2) [55].

### Differential scanning fluorimetry (Thermofluor)

The stability of the proteins was checked with a thermofluorimetric assay. For the reaction, we used 1–1 mg·mL^−1^ protein concentrations and 5× SYPRO Orange in a 25 μL volume in Hard‐Shell® 96‐Well PCR Plates (Bio‐Rad, Hercules, CA, USA). The thermocycling program ran in the SYBR Channel in the Bio‐Rad CFX qPCR machine from 25 to 75 °C, raising by 0.5 °C increments. The analysis was made by the Bio‐Rad cfx maestro software.

### Activity assay

The protons released during the dUTPase catalyzed hydrolysis of dUTP into dUMP and PPi were followed continuously at 559 nm at 25 °C using a Jasco V550 spectrophotometer [56]. Reaction mixtures contained 3 nm *Dr*DUT‐RDI, 3 nm *Dr*DUT‐LDI or 15 nm *Dr*DUT‐RGV enzyme in activity assay buffer (1 mm HEPES (pH 7.5), 5 mm MgCl_2_, 150 mm KCl and 40 μm Phenol Red indicator). After 1 min of pre‐incubation of the protein in the assay buffer, the reaction was started with the addition of 0–20 μm dUTP. Initial velocity was determined from the slope of the first 10% of the progress curve. The v‐S curve was fitted with a Michaelis–Menten curve fit by OriginPro 2018 (OriginLab Corporation, Northampton, MA, USA).

### Zebrafish genome sequencing

Genomic DNA was isolated from 2.5 hpf embryos using the ethanol precipitation method as described in [57]. Briefly, 2000 embryos were lysed in TNES buffer and treated with proteinase K at 50 °C overnight, then precipitated with 100% ethanol, washed and dissolved followed by a treatment with 100 μg·mL^−1^ RNase A/T1 and a subsequent phenol:chloroform extraction. 200–400 bp long DNA fragments were generated by sonication in a Diagenode Bioruptor Plus. Whole genome sequencing including the sequencing library preparation (NEBNext Ultra II DNA Library Prep Kit for Illumina (NEB, Ipswitch, MA, USA)) was ordered from iBioScience (Pécs, Hungary). Illumina sequencing was performed on NovaSeq 6000 instrument (Illumina, San Diego, CA, USA) with 2 × 151 run configuration. Raw data were checked using FastQC [58] the reads were mapped to the reference genome (version GRCz11, downloaded from https://hgdownload.soe.ucsc.edu/goldenPath/ danRer11/bigZips/) using bwa mem (version 0.7.17) [59]. These were converted to bam files, sorted and indexed using samtools (version 1.9) [60]. The locus of the dut gene was visualized and the dut variants in the sequencing data were identified in Integrated Genome Viewer (IGV) [61]. Genome sequencing data is made available from the Gene Expression Omnibus (GEO) database.

### Zebrafish husbandry and welfare

Zebrafish belonging to the wild‐type AB strain were maintained in the fish facility of the Biology Institute of ELTE Eötvös Loránd University adhering to standard protocols [57, 62]. Embryos were raised in E3 medium (5 mm NaCl, 0.17 mm KCl, 0.33 mm, CaCl_2_ and 0.33 mm MgSO_4_ in distilled water) in a 28.5 °C incubator. Animals older than 5 days post fertilization (dpf) were transferred to the fish facility where they were maintained in a standard 14/10 h light/dark cycle. Until ~2 weeks post fertilization (wpf) larvae were fed with commercially available dry food (100–200 μm Zebrafeed, Sparos). After 2 wpf larval and juvenile fish were fed using dry food with gradually increasing particle size (200–400 μm Zebrafeed, Sparos) in combination with fresh brine shrimp hatched in the facility. Adult fish from 1 month on were fed with dry food (Small Granual, Special Diets Services, product code: 824876) combined with brine shrimp. Experiments were carried out in accordance with the Hungarian Act of Animal Care and Experimentation (1998, XXVIII) and with the directive 2010/63/EU of the European Parliament and of the Council of 22 September 2010 on the protection of animals used for scientific purposes. All protocols and experimental procedures used in this work were approved by the Hungarian National Feed Chain Safety Office (PEI/001/1713‐2/2015) and the ELTE Eötvös Loránd University, Faculty of Science Animal Welfare Commission.

### *In silico* gene expression studies

Cap analysis of gene expression (CAGE) sequencing data from various developmental stages was visualized in the National Center for Biotechnology Information (NCBI) Genome Data Viewer using the inbuilt TSS tracks of the GRCz12tu zebrafish genome assembly (annotation release GCF_049306965.1‐RS_2025_04).

Transcriptomic and protein profiling data of early zebrafish embryos was investigated with the help of the online application provided by the Bazzini lab (https://simrcompbio.shinyapps.io/BazziniLab_Zebrafish_Protein_Profiling/) [63]. Genome ontology (GO) analysis based on the single‐cell sequencing data from the Daniocell dataset [64] was performed using the Seurat package in combination with clusterProfiler [65, 66]. Previously published single‐cell datasets for zebrafish ovary and testis [67, 68] were also reanalyzed using the Seurat package [65]. For data visualization, we used the ggplot2 and ggpubr packages in RStudio [69, 70, 71].

### Generating *dut* “crispant” zebrafish

Generation of “crispant” zebrafish was done as described before [72], using custom Alt‐R oligos purchased from Integrated DNA Technologies (IDT). Briefly, to prepare the *dut* Cas9 RNP mix for microinjection first, oligos were annealed by combining 1 μL of crRNA (200 μm), 2 μL of tracrRNA (100 μm), and 0.5 μL of ddH_2_O for a total volume of 3.5 μL. This mixture was incubated for 5 min at 95 °C. Subsequently, Cas9 RNPs were assembled by mixing 1 μL of Cas9 protein (10 μm), 0.7 μL of tracRNA hybrid (57 μm), and 1.3 μL of ddH_2_O to achieve a final volume of 3 μL. This combination was incubated for 5 min at a temperature of 37 °C, after which the mixture was ready for use. Two nanoliters of a combination comprising 13.3/μL sgRNA and 3.33 ng·μL^−1^ Cas9 protein (M0646T; New England Biolabs, Ipswich, MA, USA) in nuclease‐free water were injected into wild‐type (AB) embryos at the one‐cell stage.

To check the result of the treatment genomic DNA was extracted from 12 injected 2‐dpf‐old zebrafish embryos as described before [73]. Briefly, embryos were transferred to PCR tubes, excess medium was removed, and 100 μL of 50 mm NaOH was added. Samples were incubated at 95 °C for 15 min to lyse the tissue, with a brief mixing step performed near the end of the incubation. Tubes were then cooled to 4 °C, and 10 μL of 1 M Tris/HCl (pH 8.0) was added to neutralize the solution. After centrifugation, the supernatant was used directly for PCR, with 1 μL of lysate added per 25 μL reaction.

After gDNA isolation, PCR amplification was used to amplify the targeted region, followed by Sanger sequencing to assess the efficiency of genome editing. The sequencing results of P0 confirmed the success of our *dut* CRISPR‐mediated editing.

The targeted genomic sequence (chr18: 5308392–5318966(−)) was the following: 5′ACCGTAGTATCCGTGTGGAA−3′. Forward and reverse primers used to amplify the target sequence, respectively: 5′‐TGTCCTGATCTGCCTGTTTCAG‐3′ and 5′ACGGCTCCTTGTTGAAGTTGAA‐3′.

## Results and discussion

### *In silico* analysis of the zebrafish dUTPase isoforms

**Fig. 1:**
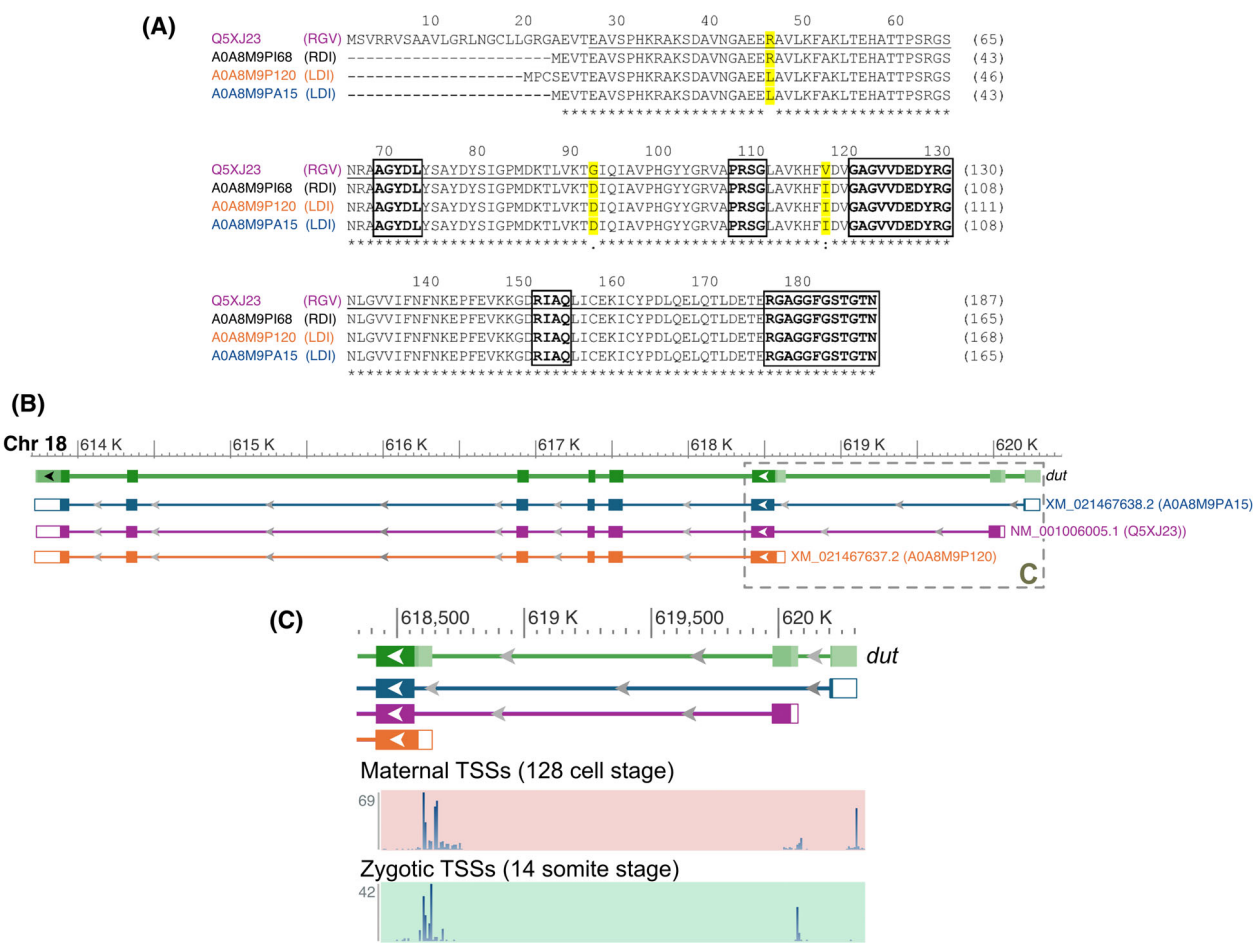
*In silico* analysis of *Danio rerio* dUTPase variants. (A) Sequence alignment of all Danio dUTPase variants each represented with their respective Uniprot code, which is colored corresponding to Panel B. Asterisk (*) denotes identity; strongly or weakly similar amino acids are marked with colon (:) and period (.) respectively. Positions of the variable residues are highlighted in yellow. The five conserved motifs are boxed. The protein variants corresponding to the underlined sequence lacking the N‐terminal extension were expressed. (B) The genomic location of the *dut* gene (green) on chromosome 18 and the exon composition of the three transcripts (named and colored as on Panel A) where active transcription can be detected. (C) Transcriptional start sites (TSSs) of the maternal and zygotic transcripts. Panels B and C were generated by Integrated Genome Viewer (IGV) [61].

There are currently four isoforms of zebrafish dUTPase (*Dr*DUT) listed in the UniProt database (https://www.uniprot.org/). Sequence alignment of these four variants shows that they differ mostly in the N‐terminus (Fig. 1A). The longest isoform (Q5XJ23) has an extended N‐terminal segment, which might correspond to a mitochondrial localization signal. Comparative analysis with the human and mouse dUTPases revealed that the zebrafish nuclear isoform shares a high degree of similarity with its mammalian counterparts, whereas the mitochondrial leader sequence exhibits pronounced divergence among the three species (cf. Fig. S2). Mapping the transcriptional start sites (TSSs) of isoforms NM_001006005.1 (encoding Q5XJ23), XM_021467637.2 (encoding A0A8M9P120) and XM_021467638.2 (encoding A0A8M9PA15), shows that the main source of variation at the N‐terminus is due to alternative splicing (Fig. 1B), similarly to the human dUTPase [74, 75]. CAGE sequencing can also be used to identify *dut* TSSs both in the maternal and zygotic transcriptome. Interestingly, we can observe that the TSS at exon 1 of *dut* can be identified only in the maternal transcriptome (Figs 1C and S3), suggesting that XM_021467638.2 is only maternally expressed. Of note, in the case of the other two transcripts, expressed both maternally and zygotically, a shift from maternal to zygotic promoters can be observed (Fig. 1C) as previously described [76].

Otherwise, the isoforms differ at three amino acid positions R46L, G92D, V117I (numbering is according to the longest isoform, i.e., Q5XJ23). From the potential eight variations of these three sites only RGV, RDI and LDI are present (as shown in Fig. 1A, variable positions are highlighted in yellow).

In *Saccharomyces cerevisiae* and *Escherichia coli*, single point mutations in the dUTPase active site (G82S in motif 3 and G147D in motif 5, respectively) abolished enzymatic activity, thereby significantly impairing the viability of the respective organisms. Thus, we checked if the variations in *Dr*DUT dUTPases are located in the conserved regions and found that none of those fall within the conserved motifs (Fig. 1).

To check the position of these variable residues within the three‐dimensional protein structure, we created structural models by AlphaFold3 [47]. The models show high structural similarity of the isoforms apart from the N‐terminal segments, which are seemingly flexible as in the case of other dUTPases (Fig. S4). Importantly, the variant residues are contained within the well‐folded part of the enzyme structure.

**Fig. 2:**
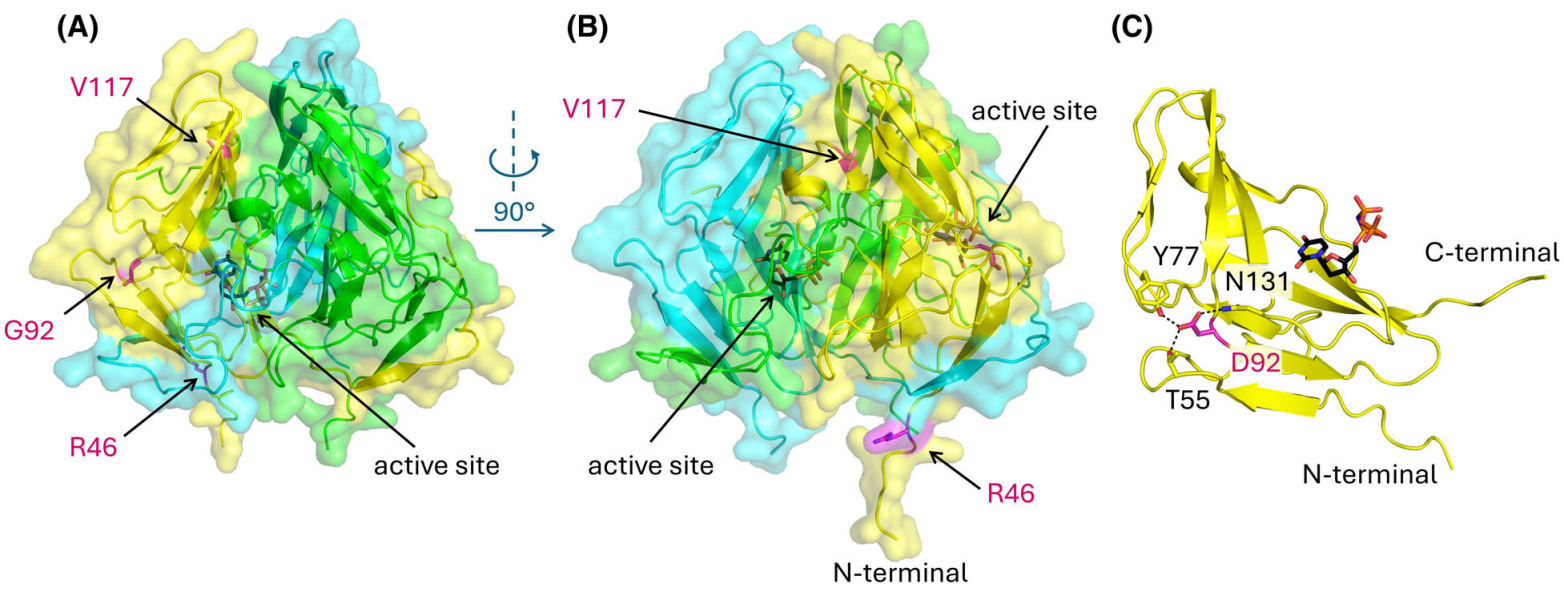
Spatial arrangement of *Danio rerio* dUTPase mutations relative to the surface and the active site of the protein. (A, B) Surface of *Dr*DUT‐RGV (Alphafold 3 model) is shown (partially transparent) from two different viewpoints (rotated around the y axis by 90 degrees) to allow inspection of all three positions. Protein chains are colored yellow, green and cyan, positions of the variable residues in case of Chain A (yellow) highlighted with magenta. The position of the substrate analogue (shown as sticks with atomic coloring carbon : black, oxygen : red, nitrogen : blue, phosphorus : orange) at the active site is modeled based on superimposition of *Dr*DUT‐RGV with the human dUTPase (PDB ID: 3EHW). (C) Intrachain hydrogen bonds of D92 with residues Y77, N131, and T55. The extended part of the carboxy terminus is not shown. Figure was created using pymol 2.5.4 (Schrodinger, LLC; https://www.pymol.org).

As expected, the variant R46, G92, V117 residues are distant from the active site. According to our models, residue R46 is located on the surface of the protein, whereas G92—and particularly V117—are positioned more internally (Fig. 2A,B). Thus, the replacement of R46 by leucine could cause instability due to its reduced hydrophilicity, while accommodation of the bulkier aspartic acid and isoleucine at the 92 and 117 positions could potentially cause a decrease in stability. Still, as human and other eukaryotic dUTPases also contain aspartic acid and isoleucine at the respective positions [77] (Fig. S2), these variations are not expected to dramatically interfere with protein folding. Accordingly, the respective 3D structural models for RDI and LDI variants suggest that the larger residues could be fairly accommodated in the structure. The aspartic acid even forms additional intrachain hydrogen bonds with residues T55, Y77, and N131 (Fig. 2C), which can further stabilize the structure.

Interestingly, a mutation (i.e., the T25I) of the *E. coli* dUTPase, which is far from the active site and is predicted to induce only a slight steric hindrance effect (cf. Fig. S5) was shown to lead to a drastic decrease in enzymatic activity [78]. Similarly, the seemingly benign Y54C mutation on the surface of the human dUTPase has been associated with disease due to the thermal instability of the enzyme [22, 23, 24]. Thus, we wanted to assess the effect of these variations on the stability of the *Dr*DUT isoforms.

We applied sequence‐based (MuProt [54], DDGemb [48], SAAFEC‐SEQ [49]) and structure‐based (DDMut [50], MAESTRO [51], PREMPS [52], MutPred2 [53]) predictions in which we implanted the best ranked Alphafold3 model to predict the effect of the amino acid variations in *Dr*DUT (Table 1). The predicted impact of the amino acid substitutions was rather inconclusive. To provide a reference, we analyzed the Y54C mutation in human dUTPase using the same prediction tools, which yielded consistent results indicating a destabilizing effect matching the experimental data.

**Table 1:** Results of *in silico* predictions of the effect of the Danio DUT mutations.

Abbreviation of the method	Refs	Input used	R46L	G92D	V117I	RGV vs LDI	hDUT Y54C
MuProt	[54]	Sequence	ddG = 0.01 neutral	ddG = −0.57 destabilize	ddG = −0.62 destabilize	Destabilize^a^	ddG = −1.13 destabilize
DDGemb	[48]	Sequence	ddG = 0.46 neutral	ddG = −0.36 neutral	ddG = 0.6 weakly stabilize	Neutral^a^	ddG = −1.37 destabilize
SAAFEC‐SEQ	[49]	Sequence	ddG = −0.29 destabilize	ddG = −1.09 destabilize	ddG = −0.47 destabilize	Destabilize^a^	Destabilize
DDMut	[50]	Structural model (RGV)	ddG = 0.15 stabilize	ddG = −2.71 destabilize	ddG = 0.20 stabilize	ddG = −4.04 destabilize	ddG = −1.55 destabilize
MAESTRO	[51]	Structural model (RGV)	ddG = 1.27 destabilize^b^	ddG = 0.14 destabilize^b^	ddG = −0.63 stabilize^b^	ddG = 0.26 destabilize^b^	ddG =2.47 destabilize^b^
PREMPS	[52]	Structural model (RGV)	ddG = 0.0 neutral^b^	ddG = 0.11 weakly destabilize^b^	ddG = −1.13 stabilize^b^	Stabilize^a^	ddG = 1.09 destabilize^b^
MutPred2	[53]	Structural model (RGV)	Score = 0.4 benign^c^	Score = 0.4 benign^c^	Score = 0.1 benign^c^	Benign^a^	Score = 0.71 pathogenic^c^

^a^ Effect of mutations predicted separately by the method, thus the overall result is the sum of the three effects.

^b^ In case of this method the difference in ΔG is defined differently than in the case of the others; if it is negative it indicates stabilization.

^c^ Score >0.611 the mutation is considered to be pathogenic.

### Experimental analysis of the zebrafish dUTPase isoforms

To verify the results of the *in silico* stability predictions, we decided to produce the different isoforms. First we tried to express the mitochondrial dUTPase of *Danio rerio* (Uniprot ID: Q5XJ23). Although expression of the recombinant *Dr*DUT‐mito protein in *E. coli* was successful, it was found exclusively in the insoluble fraction, likely as inclusion bodies, which impeded the purification of the protein in a soluble and functional form.

Subsequently, the mitochondrial leader sequence (residues 1–26; cf. Fig. 1) was removed from the construct, resulting in a vector encoding *Dr*DUT‐RGV. The other two protein variants, namely *Dr*DUT‐RDI and *Dr*DUT‐LDI were obtained by mutagenesis. The three‐letter codes in the variant names correspond to the amino acid residues at positions 46, 92, and 117, respectively, while the numbering is based on the mitochondrial zebrafish dUTPase sequence (UniProt ID: Q5XJ23, cf. Fig. 1). All three variants could be isolated and purified (Fig. S6).

**Fig. 3:**
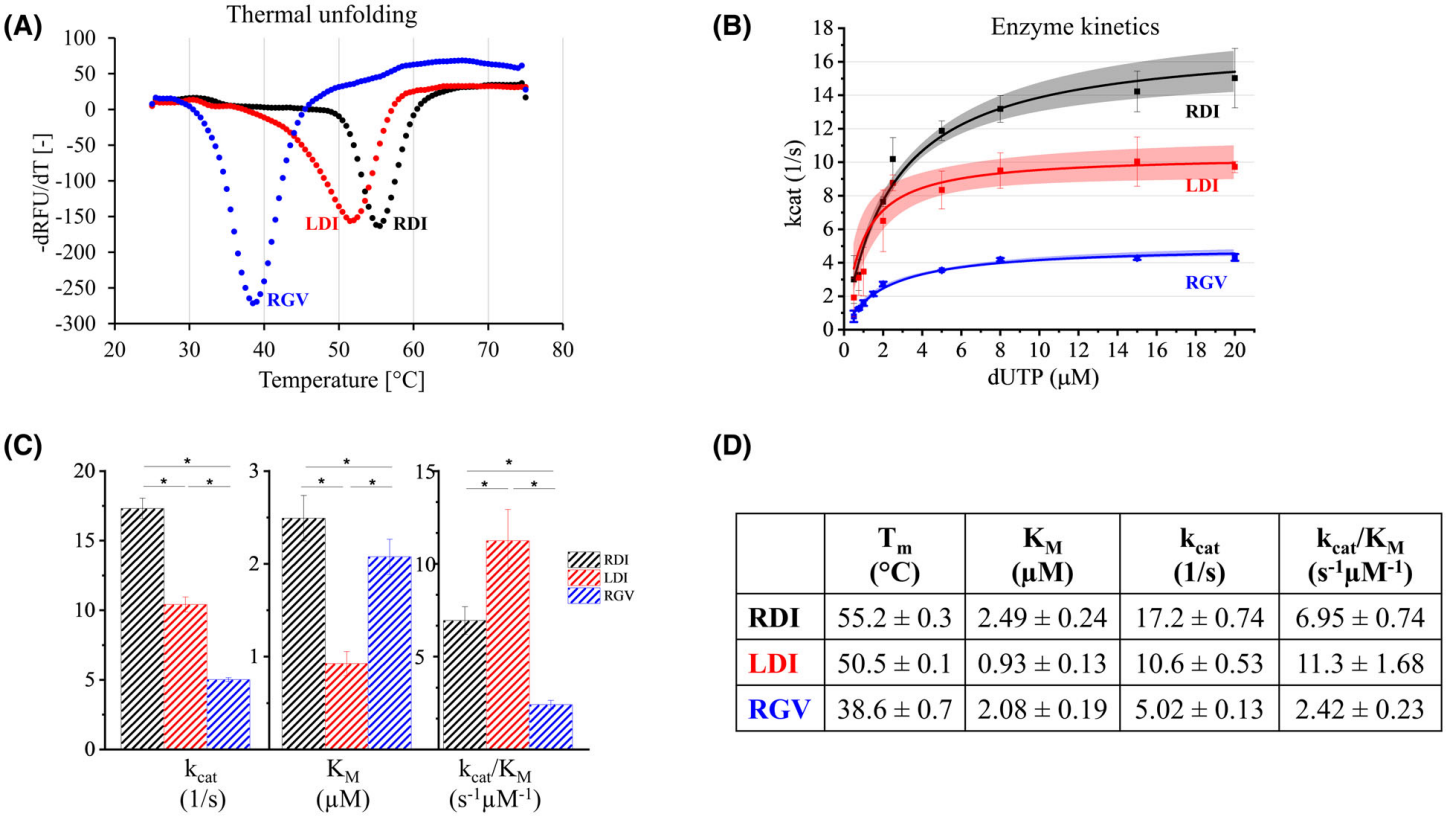
Thermal stability and catalytic activity of *Dr*DUT variants. (A) First negative derivative of the melting curves of the *Dr*DUT variants measured by differential scanning fluorimetry. Melting temperature is shown in Panel D. (B) Michaelis–Menten curves of the *Dr*DUT variants. (C, D) Numeric data of the stability and activity measurements, also showing the significance of the differences between the results (in Panel C, the *P*‐values < 0.05 from the ANOVA test are indicated by *). All measurements were done in triplicates (*n* = 3).

The thermal stability of the three variants was tested by differential scanning fluorimetry. The melting temperature (*T*_M_) of *Dr*DUT‐RGV was found to be 38.6 °C, whereas the other two variants, *Dr*DUT‐RDI and *Dr*DUT‐LDI, showed markedly improved stability associated with melting temperatures of 55.2 °C and 50.5 °C, respectively (Fig. 3A). The human dUTPase, which contains aspartate and isoleucine at the corresponding positions—thus resembling the 92D, 117I *Dr*DUT variants—exhibits a melting temperature of 58.5 ± 0.5 °C [23]. The Y54C single mutation in the human dUTPase leads to a 6 °C decrease in the *T*_M_ and was proved to cause severe physiological effects associated with the reduced thermal stability [22, 23, 24].

We tested the catalytic activity of the three *Dr*DUT variants (Fig. 3B–D). Based on our results, although the three variants have comparable affinity toward the substrate, the *K*_M_ values significantly differ between the variants. In addition, the catalytic efficiency of the three variants also differs significantly. The *Dr*DUT‐LDI variant was found to be the most active, with a *k*_cat_/*K*_M_ of 11.3 ± 1.7 s^−1^·μm ^−1^. The L46R mutation significantly reduced the catalytic activity of the protein (cf. *k*_cat_/*K*_M_ = 6.95 ± 0.74 s^−1^·μm ^−1^ of *Dr*DUT‐RDI), while the G92 and V117 variants were least active with an order of magnitude decrease in *k*_cat_/*K*_M_ (cf. *k*_cat_/*K*_M_ = 2.4 ± 0.2 s^−1^·μm ^−1^ of *Dr*DUT‐RGV). By comparison the Y54C mutation in the human dUTPase did not affect the activity of the protein [23].

In conclusion, our results show significant differences in the three variant proteins with respect to thermal stability and catalytic properties where the RGV variant is inferior to the others in both aspects, while the substrate affinity remains similar between the variants.

Our next goal was to investigate the role of dUTPase in the zebrafish animal model. Considering the dramatic differences, especially in the catalytic properties of the *Dr*DUT variants with different SNPs, it was highly important to determine which of these variants are encoded in the *Danio rerio* strain in our laboratory, since this will be used in the further experiments.

Therefore, we analyzed the whole genome sequencing data, obtained on our laboratory zebrafish strain (Fig. S7). We found that the *dut* gene in this strain encodes one of the most potent (LDI) variants associated with superior catalytic and stability parameters. This finding establishes our laboratory strain as a valid and appropriate model to study the physiological role of dUTPase in fish development.

### Consequences of dUTPase loss of function in zebrafish development

**Fig. 4:**
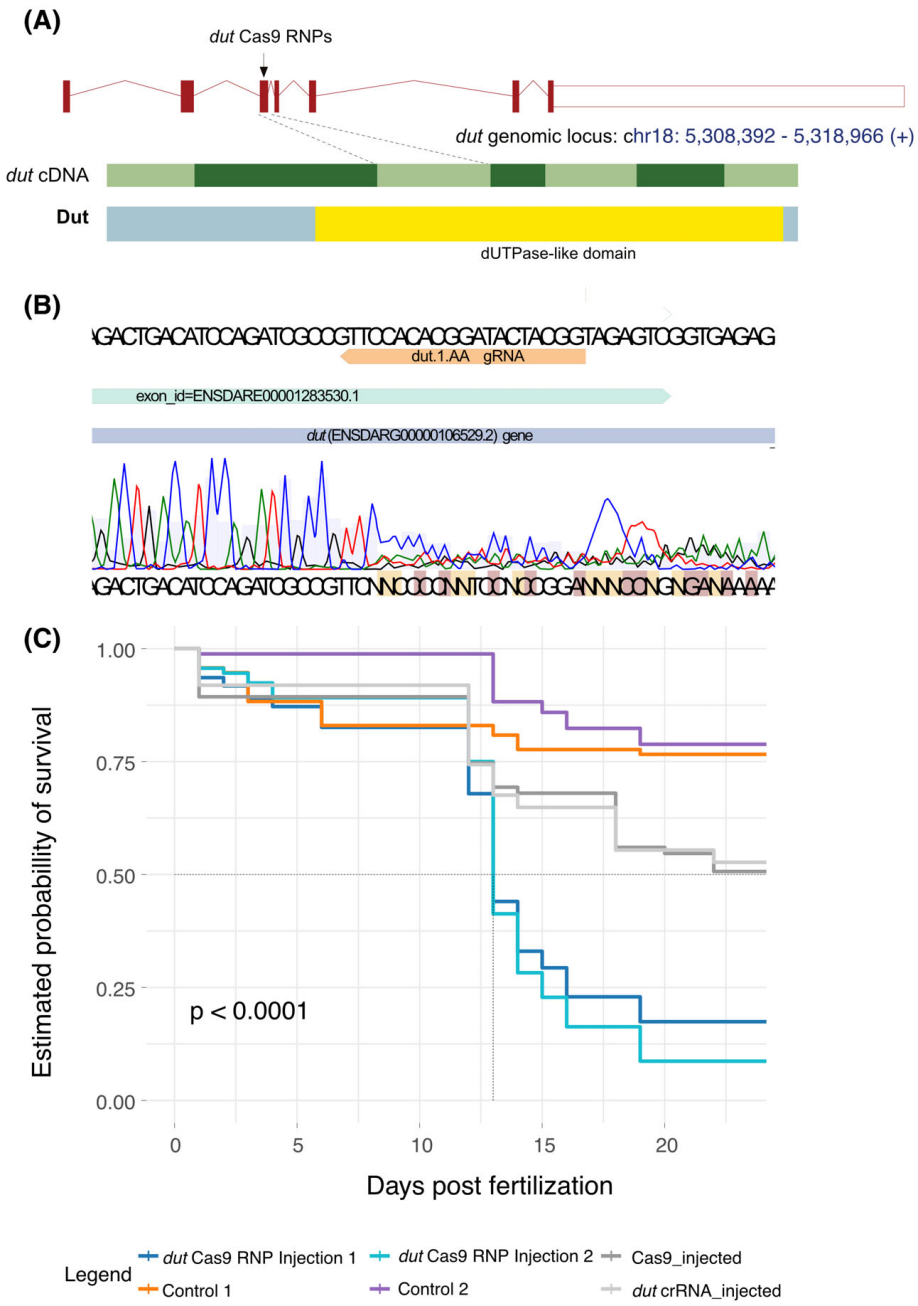
Generation and survival analysis of *dut* “crispant” zebrafish. (A) Schematic view of the zebrafish *dut* locus, *dut* transcript and the domain composition of the Dut protein. The targeting region of the *dut* sgRNA is highlighted. (In the genomic scheme red boxes denote exons; in the transcript view, light and dark green boxes highlight alternating exons, whereas in the schematic Dut view the yellow box denotes the dUTPase domain.) (B) The Sanger sequenogram of the target region using *dut* “crispant” genomic DNA from 12 embryos as a template. (C) Disruption of zygotic *dut* function results in embryonic lethality. Injection of Cas9 ribonucleoproteins (RNPs) targeting *dut* had severe effects on the estimated probability of survival of the injected embryos in two separate experiments (Group 1 and Group 2) as shown by a Kaplan–Meier survival plot. The log‐rank test was used to calculate the *p*‐value.

To better understand the role of *dut* during embryonic development we also created *dut* “crispant” zebrafish [72]. We have targeted exon 3 of the gene encoding the dUTPase domain of the enzyme (Fig. 4A) with CRISPR/Cas9 RNPs and we were able to observe highly efficient targeting activity (Fig. 4B).

While we did not observe gross morphological defects in our loss‐of‐function embryos (Fig. S8), the survival rate of the genome‐edited fish was dramatically lower than that of their non‐edited counterparts (Fig. 4C and Table 2). A high wave of mortality was observed after ~12 dpf in the “crispants,” which coincides with the early stages of metamorphosis in this species [79, 80]. This crucial phase in the life of fish is characterized by increased sensitivity to a variety of stressors [80]. While our understanding is still incomplete of this critical phase in the life of fish, available data indicate that metamorphosis is also characterized by a shift from innate to adaptive immunity [81] and the thyroid hormone‐mediated remodeling of multiple organs is also thought to be linked to new waves of proliferation [82]. As the absence of dUTPase would result in a build‐up of cellular dUTP levels, this metamorphic wave of proliferation could lead to increased levels of genomic uracil, triggering widespread cell death, ultimately compromising the survival of the animals.

**Table 2:** Survival rates of zebrafish embryos injected with *dut* Cas9 RNPs and various controls.

	Day 1	Day 6	Day 12	Day 24
Control_1	96%	83%	83%	77%
*dut RNP* inj_1	94%	83%	68%	17%
Control_2	99%	99%	99%	79%
*dut RNP* inj_2	96%	89%	75%	9%
Cas9_inj	89%	89%	75%	51%
*dut* crRNA inj	92%	92%	74%	54%

In light of these results, it was of interest to consult the existing mRNA and protein expression databases for zebrafish to see whether expression profiles are in line with the important role of dUTPase in development.

### *In silico* analysis of *dut* expression

A string of recently published datasets also made it possible for us to characterize the expression of *dut* during embryogenesis and germ cell formation in great detail.

**Fig. 5:**
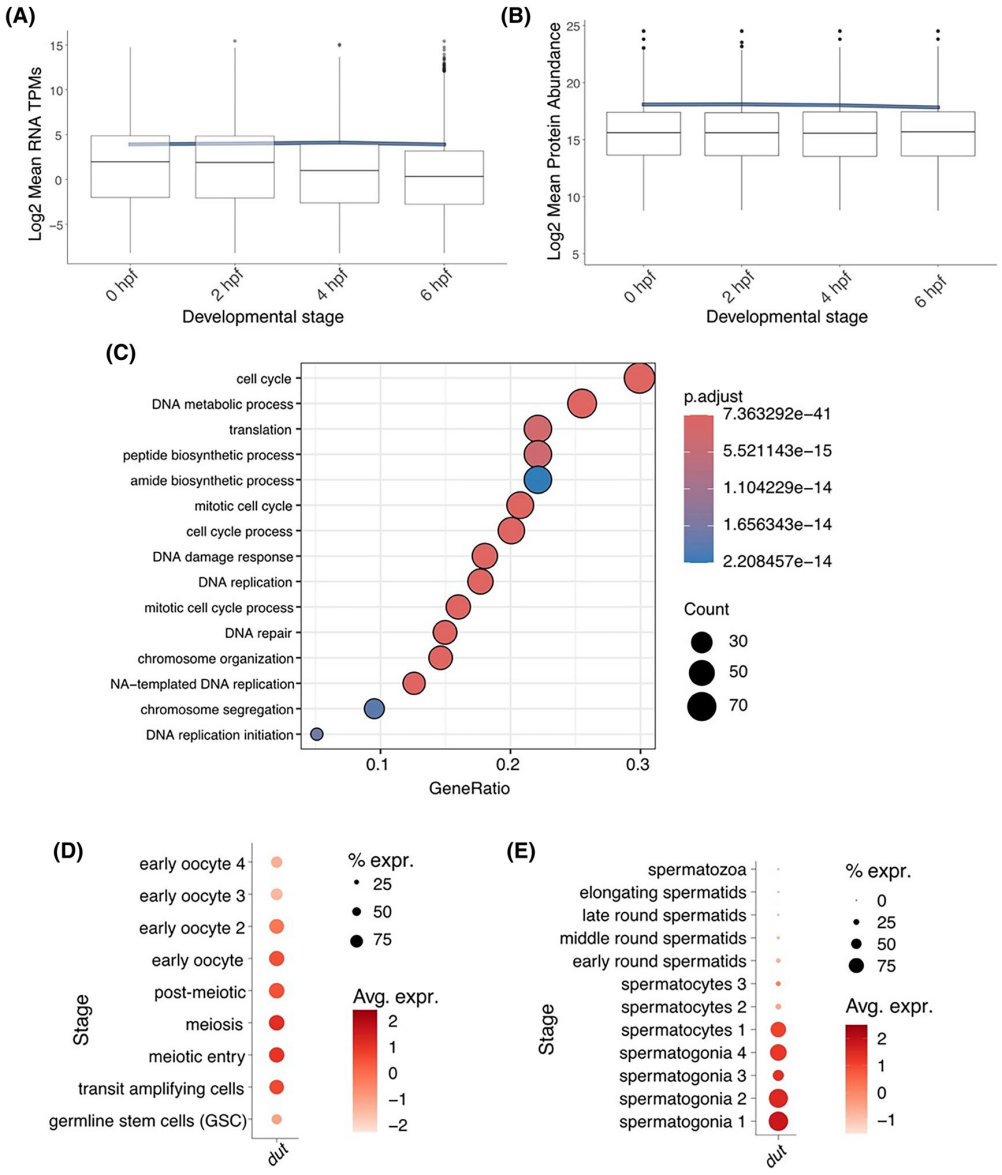
*In silico* analysis of *dut* expression during zebrafish embryogenesis and germ cell development. (A, B) Expression levels of *dut* transcripts and dUTPase protein shown as boxplot reflecting the median, interquartile range (IQR), and whiskers representing 1.5 × IQR compared to normalized means (blue) around the MTZ (3 hpf). Graph is created by Shiny script described in ref [63]. (TPM stands for “Transcripts per million”; protein abundance reflects normalized values of mass spectrometry measurements of three (Panel A) or four (Panel B) biological replicates; all data from: [63]). (C) GO analysis of genes that are significantly coexpressed with *dut* from 4 hpf to 120 hpf. (Based on data from: [64]). GeneRatio represents the proportion of genes that are coexpressed with *dut* and are annotated to a specific GO term, whereas Count represents the raw number of genes annotated to the GO term. The p.adjust value reflects the adjusted p value for the enrichment, using the Benjamini–Hochberg adjustment method. (D, E) Expression of *dut* during various stages of oogenesis (D) and spermatogenesis (E). (Based on data from: [67, 68].) The percentage of cells in the dataset expressing the gene (% expr) is shown, as is the average expression level (Avg. expr.) of the gene across all the cells in a given subgroup, calculated based on the normalized expression of the genes in the dataset.

Analysis of transcriptomic and protein profiling datasets that cover the period of MZT [63] shows that both *dut* transcripts and dUTPase protein are present maternally in the zebrafish egg, at stable concentrations throughout the earliest stages of embryogenesis (Fig. 5A,B). The reanalysis of the Daniocell single‐cell RNA sequencing (scRNA‐Seq) dataset, which covers the post‐MZT period, until 120 hpf, showed that dUTPase is expressed in a variety of tissues until ~48 hpf, in line with previously published high‐throughput expression analysis datasets [83]. To assess the functional associations of *dut* in the developing zebrafish, we performed GO enrichment analysis on genes significantly coexpressed with *dut* across all cell types. This analysis showed that coexpressed genes are significantly enriched for biological processes involved in DNA replication and cell cycle (Fig. 5C), consistent with the known role of *dut* in nucleotide metabolism. The list of genes shown in Fig. 5C is provided as a Data S1.

Finally, we were also able to follow the level of dut transcripts during oogenesis [67] and spermatogenesis [68]. Detailed reanalysis of scRNA‐Seq data suggests that while dut is present through all stages of oocyte development (Fig. 5D), its levels are only elevated during the early stages of spermatogenesis, and *dut* transcript levels decline in late spermatocytes (Fig. 5E).

The expression data supports the potential importance of dUTPase in embryogenesis along with our experimental findings. It is also important to emphasize that in our “crispant” model, the maternal effect is still present, such that oocytes provide dUTPase to the early embryos. We clearly show that later development of the offspring individuals is highly perturbed without dUTPase.

## Conclusion

The present results show that disruption of the *dut* gene in zebrafish leads to strong lethality during the post‐embryonic development of the animal. Interestingly, the onset of lethality is observed during the larval stage corresponding to early steps of metamorphosis [79, 80].

A limitation of our study in this respect is that our currently applied method does not perturb the maternal effect; hence, we can only investigate the effect of the *dut* gene disruption at the later stages when maternal supply is not present. Still, our observations clearly indicate the importance of dUTPase function for the development and viability of zebrafish, a useful model in animal studies. Our current data pave the way toward more detailed further studies focusing on the physiological role of dUTPase in vertebrate development.

We also show that a few single point mutations in the zebrafish dUTPase protein lead to severe reductions in enzymatic activity and protein stability, as reflected in enzyme kinetics and thermal stability experiments. Notably, a similar change in the case of the human dUTPase is associated with serious physiological consequences. These zebrafish dUTPase protein mutations are present in the UniProt database as the result of a single‐nucleotide polymorphism. Our results are notable since the sites of the mutations are not in the vicinity of the active site and also do not constitute a key segment in the protein core. Still, catalytic efficiency is decreased by an order of magnitude and protein stability is also highly perturbed, characterized by a decrease in the thermal unfolding pattern of the protein. The results underline the general importance of direct investigations of the potential structural and functional effects of SNPs even if those are not present at active sites or other ligand binding sites.

## Conflict of interest

The authors declare no conflict of interest.

## Author contributions

VP‐S, AB, KN, MV, BGV contributed to study design; VP‐S, LK, JM contributed to performed experiments; VP‐S, AB, KN, MV, BGV contributed to interpreted data; VP‐S, KN, MV, BGV contributed to wrote article.

## Supporting information


**Fig. S1.** Structure of the active site of human dUTPase (PDB: 3EHW).
**Fig. S2.** Sequence alignment of the mithocondrial (A) and nuclear (B) isoforms of the zebrafish, human and mouse dUTPases.
**Fig. S3.** Developmental shift in the usage of *dut* TSS sites.
**Fig. S4.** (A) Alignment of Alphafold 3 models of the four zebrafish dUTPase isoforms.
**Fig. S5.** Structural modelling of the effect of T25L mutation of the *E. coli* dUTPase.
**Fig. S6.** SDS‐PAGE gels of the purified *Dr*DUT constructs.
**Fig. S7.** Sequence of the dUTPase of zebrafish in our laboratory.
**Fig. S8.** Representative images of various control and *dut* Cas9 RNP injected zebrafish embryos at 1 dpf and 2 dpf.

**Table S1.**Mutagenesis primers used.
**Table S2.** Sequence and extinction coefficient of the expressed proteins.

## Data Availability

The data that support the findings of this study are openly available in NCBI's Gene Expression Omnibus and are accessible through https://www.ncbi.nlm.nih.gov/geo/query/acc.cgi?acc=GSE307307, GEO Series accession number [GSE307307].
