# Supplementary material for: dUTPase is essential in zebrafish development and possesses several single‐nucleotide variants with pronounced structural and functional consequences
Source: FEBS Open Bio. 2025 Dec 16;16(6):1087–101. doi: 10.1002/2211-5463.70176 (PMC13238773; doi:10.1002/2211-5463.70176)
Supplement: Supplementary file 1 — Fig. S1. Structure of the active site of human dUTPase (PDB: 3EHW). Fig. S2. Sequence alignment of the mithocondrial (A) and nuclear (B) isoforms of the zebrafish, human and mouse dUTPases. Fig. S3. Developmental shift in the usage of dut TSS sites. Fig. S4. (A) Alignment of Alphafold 3 models of the four zebrafish dUTPase isoforms. Fig. S5. Structural modelling of the effect of T25L mutation of the E. coli dUTPase. Fig. S6. SDS‐PAGE gels of the purified DrDUT constructs. Fig. S7. Sequence of the dUTPase of zebrafish in our laboratory. Fig. S8. Representative images of various control and dut Cas9 RNP injected zebrafish embryos at 1 dpf and 2 dpf. [file FEB4-16-1087-s002.pdf]

**SUPPORTING INFORMATION**

# dUTPase is essential in zebrafish development and possesses several single nucleotide variants with pronounced structural and functional consequences

Viktória Perey-Simon[1,2], Angéla Békesi[1,2], Latifa Kazzazy[3], Máté Varga[3], Beáta Vértessy[1,2,*], Kinga Nyíri[1,2,*]

[1] Department of Applied Biotechnology and Food Science, Faculty of Chemical Technology and Biotechnology, Budapest University of Technology and Economics, Műegyetem rkp. 3., H-1111 Budapest, Hungary

[2] Institute of Molecular Life Sciences, HUN-REN Research Centre for Natural Sciences, Magyar tudósok krt 2. H-1117 Budapest, Hungary

[3] Department of Genetics, ELTE Eötvös Loránd University, Pázmány Péter sétány 1/A, H-1117 Budapest, Hungary

* To whom correspondence should be addressed. Email: nyiri.kinga@vbk.bme.hu; vertessy.beata@vbk.bme.hu

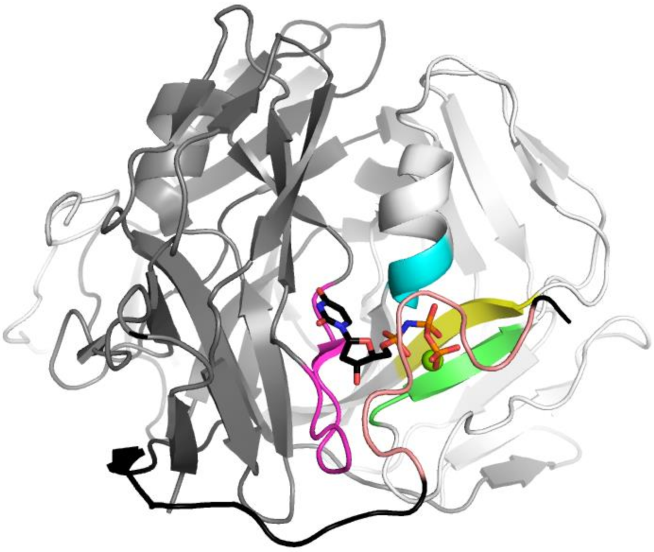

**Figure S1. Structure of the active site of human dUTPase (PDB: 3EHW).** The trimeric dUTPase is shown as cartoon, chains colored as white, grey and black. The conserved motifs are colored as: Motif 1 - green, Motif 2 - blue, Motif 3 - magenta, Motif 4 - yellow, Motif 5 - salmon. The substrate analogue dUPNPP is shown as sticks with atomic coloring (C:black, O:red, N:blue, P:orange), magnesium ion is represented as green sphere. Figure was created using PyMOL 2.5.4 (Schrodinger, LLC; https://www.pymol.org)

**A**

```
Q5XJ23-RGV      ---------------------------------------------------------MSV    3
hDUT-M          MTPLCPRPALCYHFLTSLLRSAMQNARGARQRAEAAVLSGPGPPLGRAAQHGIPRPLSSA   60
mouseDUT-M      -----------MPLLSVLLRARLQ----------AALLRGR--AL--------------G   23

Q5XJ23-RGV      RRVSAAV--------------LGRLNGCLLGRGAEVTEAVSPHKRAKSDAVNGAEERAVL   49
hDUT-M          GRLSQGCRGASTVGAAGWKGELPKAGGSPAPG--PETPAISPSKRARPAEV----GGMQL  114
mouseDUT-M      SARSRSCRGSRG---------------APAGSARADAAAVSASKR--ARAE----DGASL   62
                   * .                     .        : *:*  **              *

Q5XJ23-RGV      KFAKLTEHATTPSRGSNRAAGYDLYSAYDYSIGPMDKTLVKTGIQIAVPHGYYGRVAPRS  109
hDUT-M          RFARLSEHATAPTRGSARAAGYDLYSAYDYTIPPMEKAVVKTDIQIALPSGCYGRVAPRS  174
mouseDUT-M      RFVRLSEHATAPTRGSARAAGYDLFSAYDYTISPMEKAIVKTDIQIAVPSGCYGRVAPRS  122
                :*.:*:****:*:*** *******:*****:* **:*::***.****:* * ********

Q5XJ23-RGV      GLAVKHFVDVGAGVVDEDYRGNLGVVIFNFNKEPFEVKKGDRIAQLICEKICYPDLQELQ  169
hDUT-M          GLAAKHFIDVGAGVIDEDYRGNVGVVLFNFGKEKFEVKKGDRIAQLICERIFYPEIEEVQ  234
mouseDUT-M      GLAVKHFIDVGAGVIDEDYRGNVGVVLFNFGKEKFEVKKGDRIAQLICERISYPDLEEVQ  182
                ***.***:******:*******:***:***.** ***************:* **:::*:*

Q5XJ23-RGV      TLDETERGAGGFGSTGTN    187
hDUT-M          ALDDTERGSGGFGSTGKN    252
mouseDUT-M      TLDDTERGSGGFGSTGKN    200
                :**:****:*******.*
```

**B**

```
A0A8M9PI68-RDI      ---MEVTEAVSPHKRAKSDAVNGAEERAVLKFAKLTEHATTPSRGSNRAAGYDLYSAYDY       57
A0A8M9P120-LDI      MPCSEVTEAVSPHKRAKSDAVNGAEELAVLKFAKLTEHATTPSRGSNRAAGYDLYSAYDY       60
A0A8M9PA15-LDI      ---MEVTEAVSPHKRAKSDAVNGAEELAVLKFAKLTEHATTPSRGSNRAAGYDLYSAYDY       57
hDUT-N              MPCSEETPAISPSKRARPAEVGGM----QLRFARLSEHATAPTRGSARAAGYDLYSAYDY       56
mouseDUT-N          MPCSEDAAAVSASKRARAE--DGA----SLRFVRLSEHATAPTRGSARAAGYDLFSAYDY       54
                        * : *:*  ***:    .*      *:*.:*:****:*:*** *******:*****

A0A8M9PI68-RDI      SIGPMDKTLVKTDIQIAVPHGYYGRVAPRSGLAVKHFIDVGAGVVDEDYRGNLGVVIFNF      117
A0A8M9P120-LDI      SIGPMDKTLVKTDIQIAVPHGYYGRVAPRSGLAVKHFIDVGAGVVDEDYRGNLGVVIFNF      120
A0A8M9PA15-LDI      SIGPMDKTLVKTDIQIAVPHGYYGRVAPRSGLAVKHFIDVGAGVVDEDYRGNLGVVIFNF      117
hDUT-N              TIPPMEKAVVKTDIQIALPSGCYGRVAPRSGLAAKHFIDVGAGVIDEDYRGNVGVVLFNF      116
mouseDUT-N          TISPMEKAIVKTDIQIAVPSGCYGRVAPRSGLAVKHFIDVGAGVIDEDYRGNVGVVLFNF      114
                    :* **:*::********:* * ***********.**********:*******:***:***

A0A8M9PI68-RDI      NKEPFEVKKGDRIAQLICEKICYPDLQELQTLDETERGAGGFGSTGTN       165
A0A8M9P120-LDI      NKEPFEVKKGDRIAQLICEKICYPDLQELQTLDETERGAGGFGSTGTN       168
A0A8M9PA15-LDI      NKEPFEVKKGDRIAQLICEKICYPDLQELQTLDETERGAGGFGSTGTN       165
hDUT-N              GKEKFEVKKGDRIAQLICERIFYPEIEEVQALDDTERGSGGFGSTGKN       164
mouseDUT-N          GKEKFEVKKGDRIAQLICERISYPDLEEVQTLDDTERGSGGFGSTGKN       162
                    .** ***************:* **:::*:*:**:****:*******.*
```

**Figure S2.** Sequence alignment of the mithocondrial (A) and nuclear (B) isoforms of the zebrafish, human and mouse dUTPases Zebrafish dUTPase variants are represented with their respective Uniprot code. Asterisk (*) denotes identity, strongly or weakly similar amino acids are marked with colon (:) and period (.) respectively. The five conserved motifs are with bold red letters. Positions of the variable residues are highlighted with yellow.

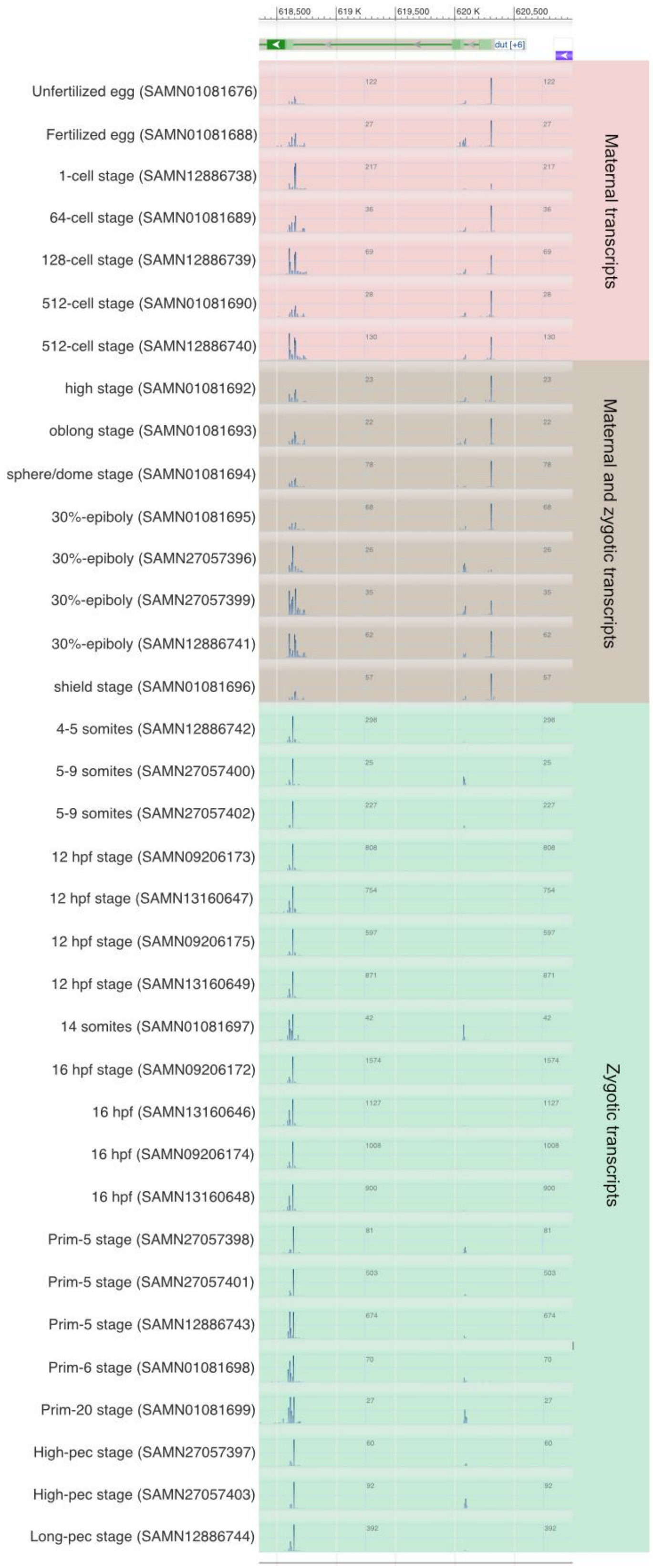


**Figure S3. Developmental shift in the usage of *dut* TSS sites**. CAGE-Seq results show that while the TSS in exon 1 is present in samples that contain solely maternal, and maternal and zygotic transcripts, in later stages, once the degradation of maternal transcripts is complete, this TSS cannot be detected anymore. For every sample the stage and the Sequence Read Archive (SRA) sample ID is shown.

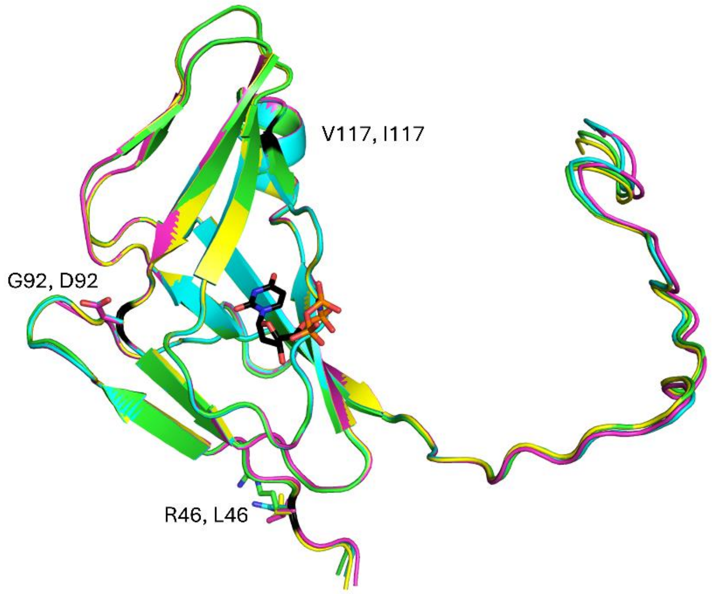


**Figure S4. A) Alignment of Alphafold 3 models of the four zebrafish dUTPase isoforms**. One chain of each isoform is shown as cartoon (Q5XJ23 with green, A0A8M9P120 with yellow and A0A8M9PA15 with magenta, A0A8M9PI68 with cyan). N-terminal residues (Residues preceding Ala-43 in Q5XJ23 and the respective residues in the other isoforms) were modeled as random coils thus not shown. The positions of the variable residues are colored black and labeled. Position of the substrate (sticks with atomic coloring as carbon: black, nitrogen: blue, oxygen: red, phosphorous: orange) is shown based on structural alignment with the human dUTPase (PDB ID: 3EHW). Figure was created using PyMOL 2.5.4 (Schrodinger, LLC; https://www.pymol.org).

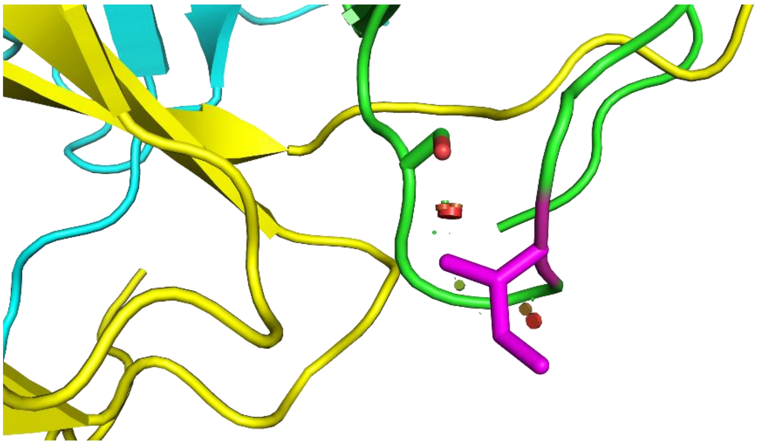

**Figure S5. Structural modelling of the effect of T25L mutation of the *E. coli* dUTPase.** The chains of the dUTPase protein are shown as cartoons, the mutated residue is shown as magenta sticks. The small red disks indicate a slight van der Waals overlap with the neighbouring residues especially with S28 (shown as sticks with atomic coloring carbon: green, oxygen: red). Figure was created using PyMOL 2.5.4 (Schrodinger, LLC; https://www.pymol.org).

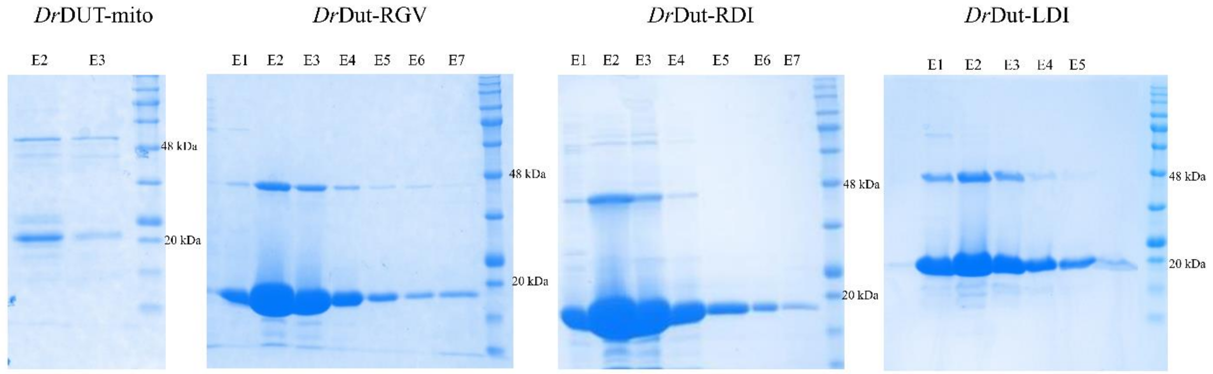


**Figure S6. SDS-PAGE gels of the purified *Dr*DUT constructs.** The E-numbers above the columns indicates the  elution fractions. The faint bands at larger molecular mass corresponds to a small fraction of covalent dUTPase dimers, which are artefatcts formed under the denaturing conditions (cf. Ref 23 in the main text).

```
1   ATG TTG GTC CGG CGT GTT TCT GCC GCT GTT TTG GGC CGT TTA AAC GGG TGT TTG CTC GGA
     M   L   V   R   R   V   S   A   A   V   L   G   R   L   N   G   C   L   L   G  20

61  CGC GGT GCT GAA GTT ACA GAA GCG GTT TCC CCG CAC AAA AGA GCC AAG AGC GAT GCG GTG
     R   G   A   E   V   T   E   A   V   S   P   H   K   R   A   K   S   D   A   V  40

121 AAC GGA GCG GAG GAG CGA GCG GTG TTA AAG TTC GCC AAG CTC ACG GAA CAC GCC ACG ACA
     N   G   A   E   E   R   A   V   L   K   F   A   K   L   T   E   H   A   T   T  60

181 CCG AGC CGA GGG TCC AAC CGG GCC GCG GGA TAC GAC CGA TAC AGT GCT TAT GAC TAC AGC
     P   S   R   G   S   N   R   A   A   G   Y   D   L   Y   S   A   Y   D   Y   S  80

241 ATC GGG CCG ATG GAC AAA ACA CTG GTC AAG ACT GAC ATC CAG ATC GCC GTT CCA CAC GGA
     I   G   P   M   D   K   T   L   V   K   T   D   I   Q   I   A   V   P   H   G  100

301 TAC TAC GGT AGA GTC GCA CCA CGA TCG GGT CTC GCT GTC AAG CAC TTC ATT GAT GTT GGT
     Y   Y   G   R   V   A   P   R   S   G   L   A   V   K   H   F   I   D   V   G  120

361 GCC GGT GTG GTT GAT GAA GAC TAC AGA GGG AAT CTG GGA GTC GTG ATC TTC AAC TTC AAC
     A   G   V   V   D   E   D   Y   R   G   N   L   G   V   V   I   F   N   F   N  140

421 AAG GAG CCG TTC GAA GTG AAG AAA GGA GAC CGC ATC GCT CAG CTC ATC TGT GAG AAG ATC
     K   E   P   F   E   V   K   K   G   D   R   I   A   Q   L   I   C   E   K   I  160

481 TGT TAC CCG GAC CTG CAG GAG TTA CAG ACG CTG GAT GAG ACG GAG CGA GGA GCG GGC GGA
     C   Y   P   D   L   Q   E   L   Q   T   L   D   E   T   E   R   G   A   G   G  180

541 TTC GGC TCC ACC GGC ACC AAC TGA
     F   G   S   T   G   T   N   *
```

**Figure S7. Sequence of the dUTPase of zebrafish in our laboratory.** DNA sequence of the dUTPase of our laboratory zebrafish line from WGS data and the corresponding protein sequence are shown, the investigated variable positions are highlighted with red.

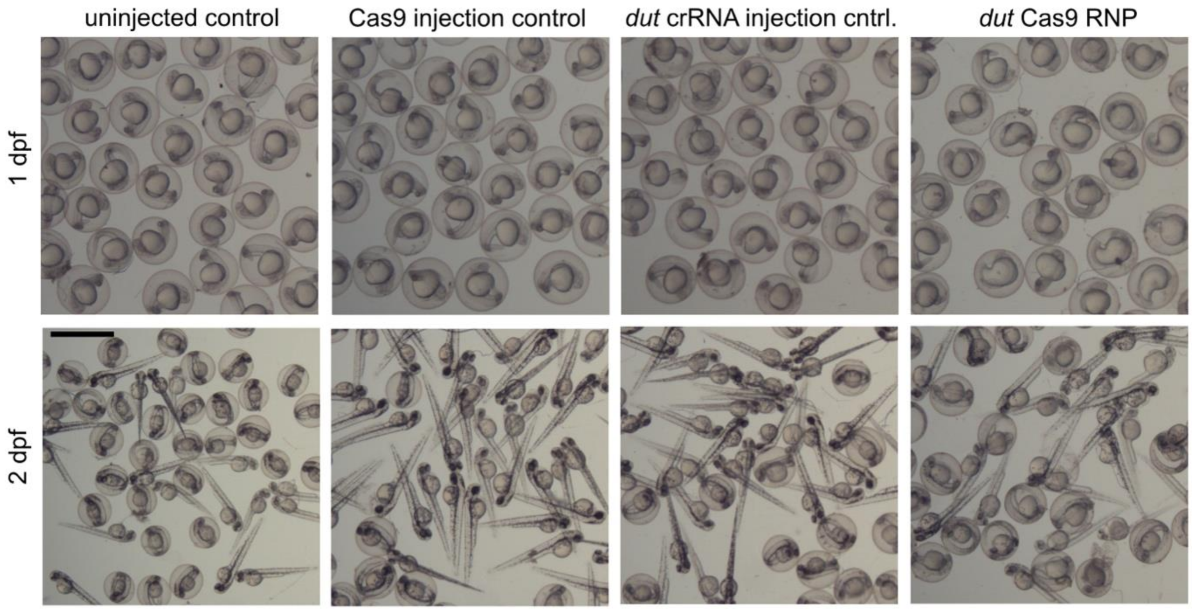


**Figure S8. Representative images of various control and *dut* Cas9 RNP injected zebrafish embryos at 1 dpf and 2 dpf.** Uninjected zebrafish embryos, embryos injected only with Cas9 protein, or *dut* crRNA were imaged together with embryos injected with *dut* Cas9 RNPs. (Scale bar, applicable to all pictures: 2 mm.)

**Table S1 Mutagenesis primers used**

| Primer name | sequence |
|---|---|
| Primer 1 | 5'-GAAGTTACCGAAGCAGTTAGTCC-3' |
| Primer 2 | 5'-GCTCATATGGCTGCCGCG-3' |
| Primer 3 | 5'-GGTTAAAACCGATATTCAGATTG-3' |
| Primer 4 | 5'-CATCAATAAAATGTTTAACTGCCAG-3' |
| Primer 5 | 5'-TTGGTGCCGGTGTTGTGGATGA-3' |
| Primer 6 | 5'-CCGAAGAACTGGCAGTTCTG-3' |
| Primer 7 | 5'-CACCATTAACTGCATCACTTTTTGC-3' |

**Table S2 Sequence and extinction coefficient of the expressed proteins**

| | Sequence | ε* |
|---|---|---|
| DrDUT-mito | MGSSHHHHHH SSGLVPRGSH MSVRRVSAAV LGRLNGCLLG RGAEVTEAVS PHKRAKSDAV NGAEERAVLK FAKLTEHATT PSRGSNRAAG YDLYSAYDYS IGPMDKTLVK TGIQIAVPHG YYGRVAPRSG LAVKHFVDVG AGVVDEDYRG NLGVVIFNFN KEPFEVKKGD RIAQLICEKI CYPDLQELQT LDETERGAGG FGSTGTN | 0.535 |
| DrDUT-RGV | MGSSHHHHHH SSGLVPRGSH MSEVTEAVSP HKRAKSDAVN GAEERAVLKF AKLTEHATTP SRGSNRAAGY DLYSAYDYSI GPMDKTLVKT GIQIAVPHGY YGRVAPRSGL AVKHFVDVGA GVVDEDYRGN LGVVIFNFNK EPFEVKKGDR IAQLICEKIC YPDLQELQTL DETERGAGGF GSTGTN | 0.592 |
| DrDUT-RDI | MGSSHHHHHH SSGLVPRGSH MSEVTEAVSP HKRAKSDAVN GAEERAVLKF AKLTEHATTP SRGSNRAAGY DLYSAYDYSI GPMDKTLVKT DIQIAVPHGY YGRVAPRSGL AVKHFIDVGA GVVDEDYRGN LGVVIFNFNK EPFEVKKGDR IAQLICEKIC YPDLQELQTL DETERGAGGF GSTGTN | 0.589 |
| DrDUT-LDI | MGSSHHHHHH SSGLVPRGSH MSEVTEAVSP HKRAKSDAVN GAEELAVLKF AKLTEHATTP SRGSNRAAGY DLYSAYDYSI GPMDKTLVKT DIQIAVPHGY YGRVAPRSGL AVKHFIDVGA GVVDEDYRGN LGVVIFNFNK EPFEVKKGDR IAQLICEKIC YPDLQELQTL DETERGAGGF GSTGTN | 0.591 |

* extinction coefficient $g^{-1}\ dm^3\ cm^{-1}$
